# Engineering nitrogen and oxygen functionalities in naturally sourced activated carbon for multicomponent gas adsorption

**DOI:** 10.1038/s41598-025-13430-4

**Published:** 2025-08-01

**Authors:** Xiupeng Cheng, Zhipeng Qie, Huaizhong Xiang, Zhongbao Liu, Limingxin Zong, Wenqi He, Xinxin Pi, Hassan Alhassawi, Peiyao Cao, Guang Yang, Shuangshuang Gao

**Affiliations:** 1 College of Mechanical and Energy Engineering, Beijing University of Technology, Beijing, 100124 China; 2 Chongqing Research Institute of Beijing University of Technology, Chongqing, 401121 China; 3 Department of Chemical Engineering, The University of Manchester, Manchester, M13 9PL UK; 4 Department of Chemistry, Queen Mary University of London, London, E1 4NS UK; 5 College of Mechanical and Electrical Engineering, Qingdao University, Qingdao, 266071 China

**Keywords:** Activated carbon (AC), Nitrogen doping, Adsorption, Carbon dioxide, Toluene, Chemical engineering, Biomaterials

## Abstract

Nitrogen doping is a widely adopted strategy to enhance the gas adsorption performance of activated carbon (AC) adsorbents. However, the simultaneous evolution of oxygen and nitrogen functional groups—especially in carbon precursors with high oxygen content—has received limited attention. In this study, coal-derived ACs with high surface areas (up to 940 m^2^/g) and micropore volumes (0.36 cm^3^/g) were synthesized via K_2_CO_3_-assisted physical activation, followed by nitrogen doping through co-pyrolysis with melamine. By regulating the doping temperature (600–900 °C), the nitrogen content of the resulting samples ranged from 1.44 to 7.68 at%, while the oxygen content varied from 6.89 to 10.39 at%. After decoupling the influences of porosity, we found that a well-balanced distribution of N and O functionalities, especially pyrrolic nitrogen, ether (C–O–C), and hydroxyl (C–O–H) groups, was critical for enhancing CO_2_ and H_2_O adsorption. NAC-600 exhibited the most favorable surface chemistry for the adsorption of CO_2_ (15 vol%) and H_2_O (20% RH), achieving capacities of 41 mg/g and 59.9 mg/g, respectively. In contrast, NAC-900, prepared at the highest N-doping temperature, exhibited the best surface chemistry for toluene adsorption (550 mg/cm^3^), attributed to its higher degree of graphitization and the presence of graphitic N and ether groups. This work offers a rational design strategy for improving the multicomponent gas adsorption performance of activated carbons for flue gas treatment.

**Supplementary Information:**

The online version contains supplementary material available at 10.1038/s41598-025-13430-4.

## Introduction

Carbon Capture, Utilization, and Storage (CCUS) has emerged as a critical research frontier in the fields of energy and environmental science, with carbon capture recognized as one of its pivotal components. Current post-combustion CO_2_ capture strategies primarily encompass chemical absorption, adsorption, and membrane separation technologies^1^. Among them, amine-based chemical absorption has been widely scaled up and implemented in industrial settings such as coal-fired power plants. However, amine solutions are prone to thermal and oxidative degradation during cyclic operation, resulting in limited recyclability and the formation of hazardous by-products such as nitrosamines, which may lead to significant environmental and health risks^2,3^. Compared to chemical absorption, adsorption using solid sorbents offers greater structural stability and requires relatively lower regeneration energy^4^, making it a promising and cost-effective technology^5,6^. Among the broad range of solid adsorbents, activated carbon (AC) has attracted enormous attention due to its abundant and low-cost precursors (e.g., coal and biomass), high specific surface area, and tunable pore configuration, making it a promising candidate for large-scale gas capture applications, such as SO_2_, CO_2_ and volatile organic compounds (VOCs)^7^.

The physical and chemical properties of ACs play a crucial role in determining their gas adsorption performance. Rehman et al.^8^ employed a one-pot polymerization–carbonization–activation method to synthesize ACs and elucidated the micropore-filling mechanism for CO_2_ adsorption (kinetic diameter, KD = 0.33 nm), highlighting the high adsorption potential of micropores with diameters below 0.73 nm. Cao et al.^9^ analyzed the linear correlation between pore structure parameters and CO_2_ capture performance, concluding that ultramicropores are the primary contributors to adsorption efficiency. In addition, it was demonstrated that an adequate presence of mesopores can also enhance CO_2_ adsorption by facilitating the diffusion of gas molecules into narrower micropores^10^, and carbon adsorbents with abundant microporosity have also shown favorable adsorption capacity for water vapor (KD = 0.28 nm)^11^. For the adsorption of gas molecules with relatively larger size (e.g., toluene, KD = 0.59 nm), although micropores remain important, Qie et al.^12^ reported that AC with a micro-mesoporous structure is more effective in the capture of toluene compared to the microporous one. Cao et al.^13^ reported a significantly enhanced toluene adsorption rate, attributed to an optimized macroporous structure that facilitates efficient mass transfer. Therefore, the appropriate matching between the pore size of adsorbents and the KD of adsorbates is critical for excellent adsorption performance.

Except for pore optimization, “defect engineering” of carbon materials has been regarded as an effective protocol to enhance their gas adsorption capability, especially the defect resulting from heteroatom doping, such as nitrogen (N). The introduction of heteroatoms can alter the distribution and transfer of electrons on the carbon surface, which may affect its affinity to the target adsorbate molecules. As for CO_2_, whose molecule has no dipole moment but a quadrupole moment, Adeela Rehman et al.^8^ demonstrated the distinct roles of various N functional groups in its capture and concluded that pyridinic, pyrrolic, and graphitic N species positively contribute to its adsorption capacity. Recently, Li et al.^14^ systematically reviewed the performances of N-doped porous carbon materials for CO_2_ adsorption and separation, and proposed that N-doping can enhance the interaction between CO_2_ and carbon adsorbents, leading to a higher CO_2_ adsorption capacity. As for toluene with weak molecular polarity, Lu et al.^15^ synthesized N-doped (high to 6.71 at%) hierarchical porous carbons with large specific surface area, presenting an improved toluene adsorption capacity compared to the one without N doping. Overall, the rational incorporation of heteroatom groups into ACs has proven to be a feasible way to achieve better gas adsorption performance.

Nitrogen-doped porous carbon, as a green and low-cost adsorbent, holds great potential for flue gas pollutant control. However, the specific adsorption preferences of different functional sites on its carbon skeleton remain insufficiently understood. For example, strong competitive adsorption is likely to occur between CO_2_ and toluene^16^. Moreover, many studies rely solely on CO_2_ and N_2_ as feed gases, often neglecting the influence of H_2_O vapor on the adsorption bed. In reality, industrial flue gas typically contains 8–15% or even higher concentrations of water vapor, which significantly affects the carbon capture process^17^. Therefore, it is of great significance to investigate the individual adsorption behaviors of CO_2_, H_2_O, and toluene, and to elucidate the surface chemical functionalities that govern their respective adsorption mechanisms. In many studies focused on the positive effect introduced by N doping, the influence of O functional groups inherited from the carbon precursor is usually ignored, especially the natural-sourced ones (e.g., coal with high O abundance). Actually, during the preparation of ACs at high temperatures (above 700 °C), the introduction of N sources (e.g., NH_3_ and melamine) can result in the partial substitution of O species, and then lead to the coexistence of N and O functional groups. This makes the surface chemistry composition of ACs more complicated, making it challenging to establish the structure–property relationships for gas adsorption. To address this issue, in this study, a series of coal-based ACs with N doping were synthesized through an activation-carbonization protocol at varying temperatures. Using characterization techniques such as XPS and FTIR, the evolution of both N and O functional groups with an increase in activation temperature was systematically revealed. Influences of the surface chemistry of ACs (i.e., N and O functionalities) on their adsorption capacities of CO_2_ and other components in flue gas (H_2_O and toluene) were investigated, providing guidance for the design and synthesis of AC adsorbents adapted to multi gas species.

## Materials and methods

### Material and pretreatments

Zhundong coal, a medium–low rank coal resourced from Xinjiang, China, was selected as the carbon precursor in this study. Its elemental and ash content analysis have been reported in our previous study^12^. Before the experiment, the coal particles (~ 150 μm size) were mixed with 5 M HCl (hydrochloric acid) solution and treated with a hydrothermal water bath at 50 °C for 12 h, aiming to remove the ash components (especially AAEMs, Alkali and Alkaline Earth Metals) in the coal. After that, the coal was washed with deionized water and dried following the steps reported in the previous study. Melamine (99%) and anhydrous potassium carbonate (K_2_CO_3_, AR) were purchased from the Shanghai MACKLIN reagent company.

### Preparation of NACs

A two-step method was employed to prepare nitrogen-doped activated carbons (NACs). The process involved an initial CO_2_-assisted activation at 850 °C, followed by co-pyrolysis with melamine. Prior to activation, Zhundong coal particles were impregnated with 3 wt% K_2_CO_3_ to promote the reaction between CO_2_ and carbon matrix at high temperatures, thereby enhancing micropore formation. Specifically, 0.06 g of K_2_CO_3_ was dissolved in 20 mL of deionized water, and 2 g of Zhundong coal particles were added to the solution. The mixture was subjected to hydrothermal treatment at 80 °C for 1 h under continuous stirring at 500 rpm. The resulting suspension was subsequently dried at 85 °C for 24 h to obtain a solid precursor. The dried mixture was then activated in a tubular furnace under a gas flow of CO_2_ (60 mL/min) and N_2_ (100 mL/min) at 850 °C for 1 h, with a ramp rate of 10 °C/min, and the prepared sample was denoted as AC. For nitrogen doping, AC was mixed with 20 mL of melamine solution (mass ratio of carbon to melamine = 2:1) and dried under the same conditions as described above. As-obtained solid mixture was then calcined at 600 °C, 750 °C, and 900 °C for 1 h under a pure N_2_ atmosphere (100 mL/min) with a ramp rate of 10 °C/min. As-obtained samples were denoted as NAC-600, NAC-750 and NAC-900, respectively.

### Characterization

The surface morphology of the NAC sample was characterized by scanning electron microscopy (SEM, ZEISS Sigma 360) at 3 kV. Pore parameters were obtained by N_2_ physisorption and desorption analyzer (Micromeritics ASAP 2460) at 77 K. Before adsorption, the AC samples were degassed at 250 °C for 5 h. The surface area was calculated according to the multi-point Brunauer–Emmett–Teller (BET) method, and the pore size distribution was obtained based on the Density functional theory (DFT) model. The surface element chemical properties and element content were acquired by X-ray photoelectron spectroscopy (XPS) on a spectrometer (Thermo Scientific K-Alpha) equipped with Al Kα radiation. The spectra were calibrated with respect to the high-resolution C 1s peak at 284.5 eV. Raman spectra of the AC samples were obtained by a Raman Spectrometer (Horiba LabRAM HR Evolution), and the exciting line was selected with a 532 nm wavelength. Fourier Transform infrared spectroscopy (FTIR) spectra were measured by an infrared spectrometer (Thermo Fisher Scientific Nicolet iS20). Before the test, AC samples were mixed with an appropriate amount of dry potassium bromide (KBr) powder, and the resulting mixture was then pressed into a transparent pellet using a tablet press. The resolution for the FTIR test was 4 cm^‒1^.

### Adsorption tests

The gas adsorption performance of the AC samples was evaluated using a Dynamic Gas/Vapor Sorption Analyzer (BSD-DVS, Beishide). Before testing, the AC samples were degassed by heating at 150 °C under a high-purity N_2_ flow (99.999%) at 400 mL/min. For a typical adsorption experiment, approximately 50 mg of AC powder was loaded into a quartz sample holder, which was connected to an ultra-high precision microbalance within the analyzer. During the adsorption process, gas flow with a total flow rate of 400 mL/min was introduced into the chamber to contact the AC powder, and the mass change was continuously recorded at a data acquisition rate of ca. 1 point/min until adsorption equilibrium was nearly reached. CO_2_ (99.999%) and toluene (500 ppm) were supplied from gas cylinders, while water vapour was introduced via a bubbling method, in which moisture was carried by the N_2_ gas flow. The adsorption capacity of adsorbate per unit mass of adsorbent (i.e., the value directed acquired by the analyzer, unit: mg/g) is denoted as *q*_m_. Adsorption capacity per unit surface area and volume of micropore were denoted as *q*_s_ and *q*_v_, respectively:

1 $$q_{{\text{s}}} = q_{{\text{m}}} /S_{{{\text{mic}}}}$$

2 $$q_{{\text{v}}} = q_{{\text{m}}} /V_{{{\text{mic}}}}$$

where *S*_mic_ and *V*_mic_ represent the specific surface area and pore volume of micropores, respectively, as determined from the N_2_ physisorption/desorption isotherms using the *t*-plot method.

### Fitting of CO_2_ adsorption isotherms

In this study, we employed two widely accepted adsorption isotherm models, i.e., Langmuir and Freundlich to fit the CO_2_ adsorption data. The Langmuir model assumes that the surface of the adsorbent is homogeneous and that adsorption occurs as a monolayer at a finite number of well-defined, localized sites, which are identical and energetically equivalent^18,19^. The Langmuir isotherm equation is given as Eq. (3)^20^.

3 $$q_{{\text{e}}} = \frac{{q_{{\text{m}}} K_{{\text{L}}} P}}{{1 + K_{{\text{L}}} P}}$$

where *q*_e_ (mg/g) is the gas adsorption capacity when the adsorption system reaches equilibrium, *P* (kPa) is the pressure, *q*_m_ (mg/g) is the maximum adsorption capacity, *K*_L_ (kPa/mg) is the Langmuir constant.

The Freundlich model is an empirical equation that describes the adsorption equilibrium on heterogeneous surfaces. The slope parameter (1/*n*), which typically ranges between 0 and 1, reflects the adsorption intensity or surface heterogeneity—the closer its value is to zero, the greater the surface heterogeneity^20^. A value of 1/*n* less than one suggests a favorable chemisorption process, whereas a value greater than one may indicate cooperative adsorption behavior^21^. Freundlich isotherm model is given as Eq. (4)^22^.

4 $$q_{{\text{e}}} = K_{{\text{F}}} P^{\frac{1}{n}}$$

where *K*_F_ (${\text{mg}}/{\text{g}}/{\text{kPa}}^{\frac{1}{n}}$) and n are the Freundlich constants related to adsorption capacity and adsorption intensity, respectively.

## Results and discussion

### Morphology and pore configuration of ACs

**Fig. 1:**
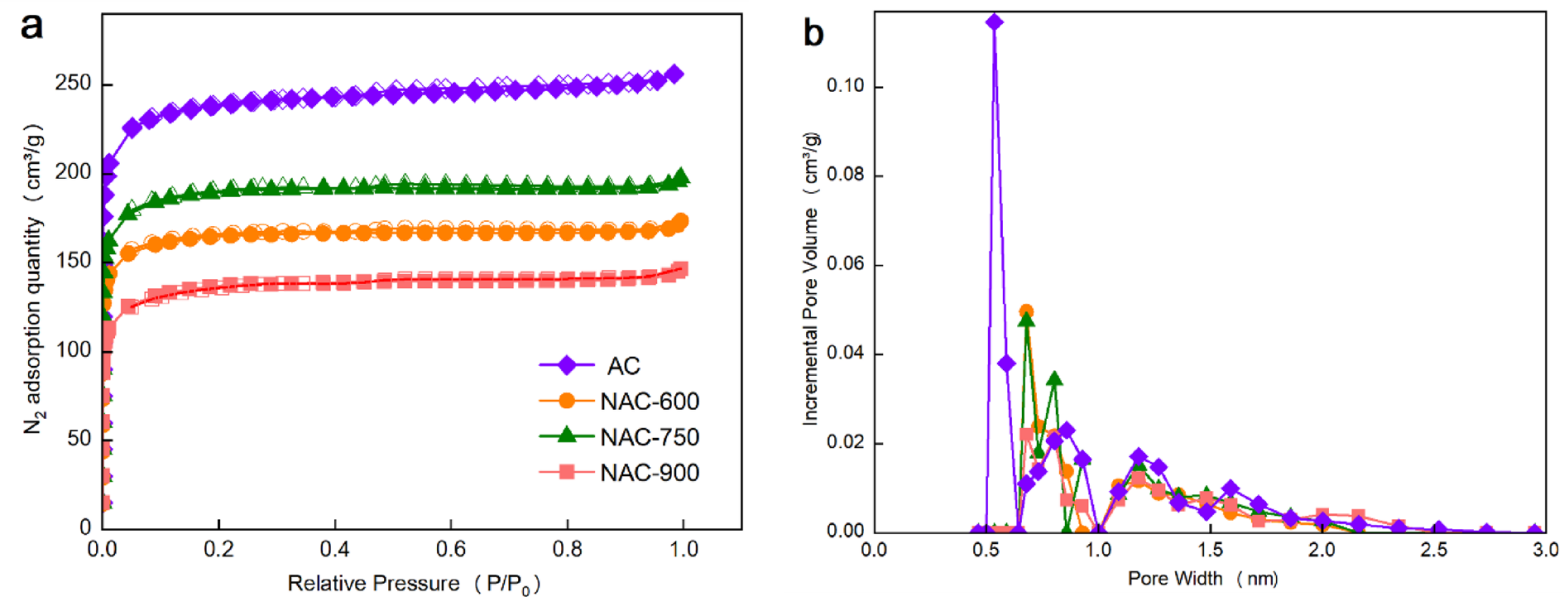
(**a**) N_2_ physisorption isotherms (@77 K) and (**b**) corresponding DFT pore size distribution profiles of the ACs.

The N_2_ physisorption isotherms of the AC samples are shown in Fig. 1a. All ACs exhibit a sharp increase in N_2_ adsorbed quantity at a relative pressure (*P/P*_*0*_) below 0.01, with no significant hysteresis loops observed. These are typical characteristics of type I adsorption isotherms, indicating the dominance of microporosity in the ACs. This is further evidenced by the high micropore volume fraction (i.e., *V*_mic_/*V*_t_) of ACs, as listed in Table 1, ranging from 0.79 to 0.92. The N_2_ adsorbed quantity at *P/P*_*0*_ = 0.01 follows the sequence: AC > NAC-750 > NAC-600 > NAC-900, corresponding to the *V*_mic_ values of 0.36 cm^3^/g, 0.26 cm^3^/g, 0.23 cm^3^/g and 0.18 cm^3^/g, respectively. A similar trend is observed for both the specific surface area *S*_BET_ and total pore volume *V*_t_. Above results suggest that the post-treatment for N doping (i.e., co-pyrolysis with melamine) led to a reduction in both *V*_t_ and *S*_BET_ compared to the pristine AC, which is resulted from the decreased accessibility of pores to gas molecules. Notably, the porosity loss is less significant when the temperature for N doping increases from 600 °C to 750 °C, due to a more sufficient decomposition of melamine (starts at 557 °C) at a relatively higher temperature. The mitigation of pore blockage makes the *V*_mic_ of sample NAC-750 higher than NAC-600. Conversely, when the temperature for N doping was further increased to 900 °C, *V*_mic_ of NAC-900 was found to severely decreased, likely attributed to the partial collapse of carbon framework at such a high temperature, even being higher than the activation temperature of AC (850 °C).

**Table 1:** Pore parameters of the ACs.

Sample	*S*_BET_^a^ (m^2^/g)	*V*_t_^b^ (cm^3^/g)	*S*_mic_ (m^2^/g)	*V*_mic_^c^ (cm^3^/g)	*D*_p_ (nm)	*V*_mic_/*V*_t_
AC	940	0. 39	914	0. 36	1. 75	0. 92
NAC-600	653	0. 27	588	0. 23	1. 64	0. 85
NAC-750	746	0. 31	665	0. 26	1. 64	0. 84
NAC-900	523	0. 23	444	0. 18	1. 73	0. 79

^a^ Calculated based on BET multi-point method, ^b^ determined by N_2_ adsorption quantity at *P*/*P*_0_ = 0. 99, ^c^ calculated based on *t*-plot method.

Figure 1b shows the pore size distribution (PSD) of ACs, highlighting the range of 0.5–3 nm, which is known as the dominant pore space for gas adsorption. All ACs exhibit two PSD peak clusters at ca. 0.75 nm and ~ 1.2 nm. The pristine AC also presents an additional sharp PSD peak at ca. 0.6 nm, which becomes absent after N-doping treatment, mainly attributed to the blockage of ultra-micropores. In contrast, the PSD peak clusters at ca. 0.75 nm of NAC-600 and NAC-750 are higher than the pristine AC. As for NAC-900, the PSD peaks in the 0.5–1 nm range are relatively lower than NAC-600 and NAC-750, further confirming the loss of ultra-micropores caused by structural collapse. Overall, K_2_CO_3_-assisted physical activation effectively produces microporous ACs, while the subsequent N-doping treatment via co-pyrolysis with melamine results in a partial blockage of micropores, especially at a relatively low temperature such as 600 °C.

**Fig. 2:**
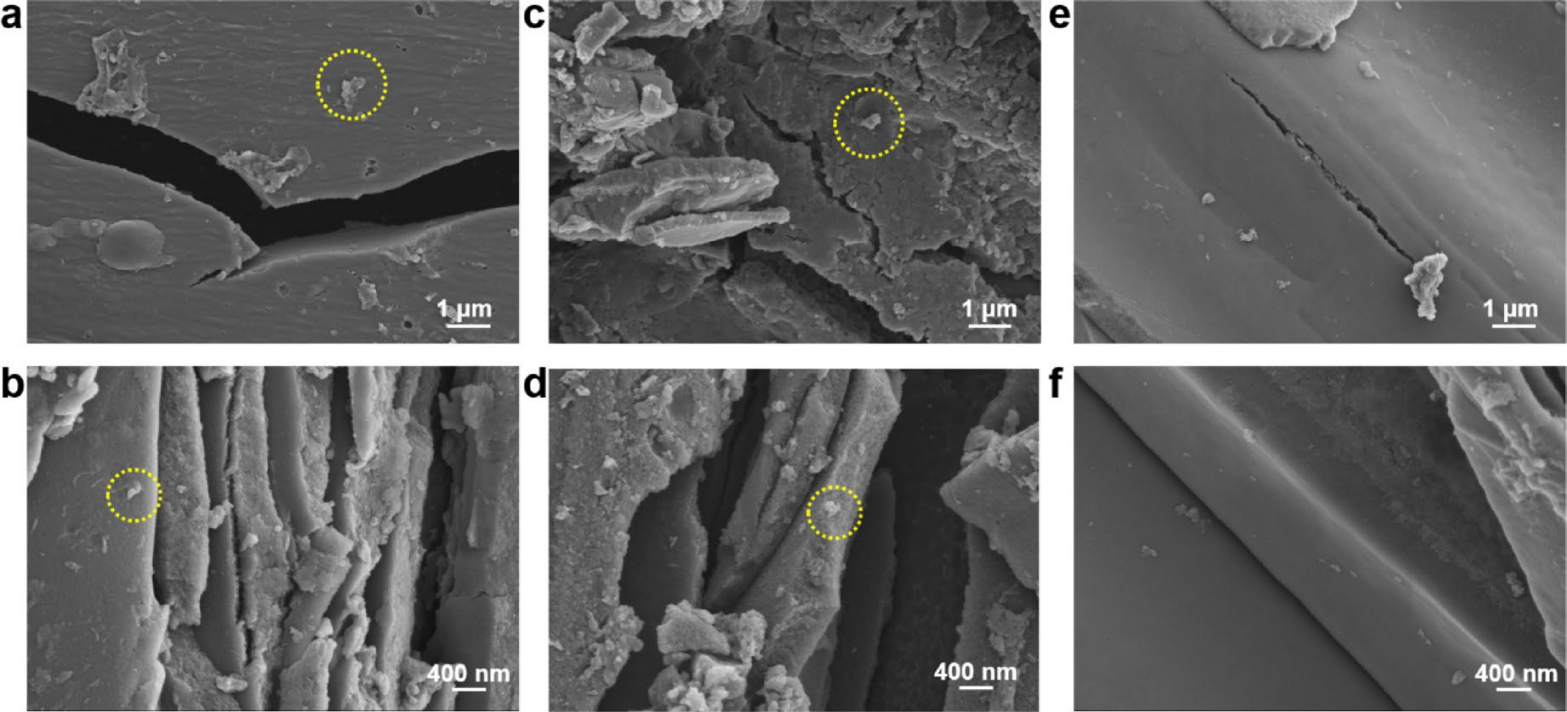
SEM images of the sample (**a**, **b**) NAC-600, (**c**, **d**) NAC-750 and (**e**, **f**) NAC-900.

Figure 2 presents the SEM images of the three NAC samples. Cracks and wrinkles, inherited from the natural carbon source (i.e., coal) can be observed on the surfaces of all three samples. Large macropores in the μm size range, combined with the micropores formed during CO_2_ activation (which are indistinctive in SEM images), form a hierarchical pore structure that facilitates gas transport and storage. Additionally, μm-scale debris aggregated on the surfaces of the NAC samples is visible, with the light-colored regions highlighted in Fig. 2a–d, attributed to residual left from melamine. With an increase in the temperature for N-doping (i.e., 750 and 900 °C), these surface features gradually disappear and become completely undetectable in NAC-900. The smooth surface observed in Fig. 2e–f is attributed to the complete pyrolysis of melamine at the temperature exceeding its decomposition point.

### Chemical properties of ACs

**Fig. 3:**
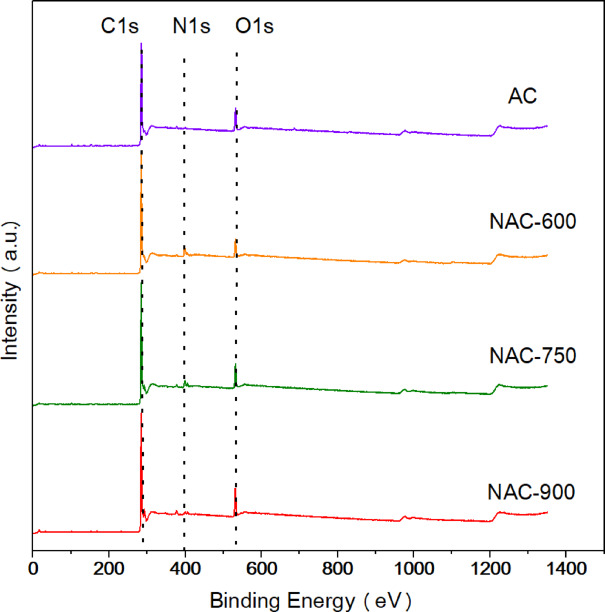
XPS survey spectra of the ACs.

XPS analysis was conducted to investigate the elemental composition and surface chemical states of the AC samples, and the survey spectra are presented in Fig. 3. The results indicate that the ACs mainly composed of C, O and element, along with trace amounts of potassium (K). The presence of K originated from the 3 wt% K_2_CO_3_ used during the activation process, as confirmed by the XPS spectra in Fig. S1. The quantitative elemental compositions of C, N, and O are summarized in Table 2. The pristine AC exhibits a relatively high O content of 10.39 at%, which can be attributed to the high O abundance in the carbon precursor used in this study, as well as a low N content (i.e., 1.44 at% in pristine AC). After N-doping at 600 °C, the N content in NAC-600 significantly increased to 7.68 at%, while the O content decreased to 6.89 at%. With a further increase in N doping temperature, the N content in NAC-750 and NAC-900 decreased to 5.55 at% and 3.13 at%, respectively, whereas the O content increased to 8.92 at% and 10.09 at%, respectively. These results indicate that the introduction of N functional groups onto coal-based AC surfaces enables the simultaneous regulation of both N and O content. N-doping at moderate temperatures enables a balanced incorporation of N and O functional groups. However, at excessively high temperatures (e.g., 900 °C), N functional groups introduced by melamine tend to decompose, leaving some thermally stable functionalities, such as ethers and epoxides. Hence, these O functional groups become dominant again in NAC-900.

**Table 2:** Element content of the ACs acquired from XPS.

Sample	C (at%)^a^	N (at%)^a^	O (at%)^a^	N (wt%)^b^
AC	87. 22	1. 44	10. 39	–
NAC-600	84. 94	7. 68	6. 89	8.64
NAC-750	84. 64	5. 55	8. 92	6.24
NAC-900	85. 14	3. 13	10. 09	3.52

^a^Measure by XPS; ^b^measured by combustion coupled with TCD detector.

**Fig. 4:**
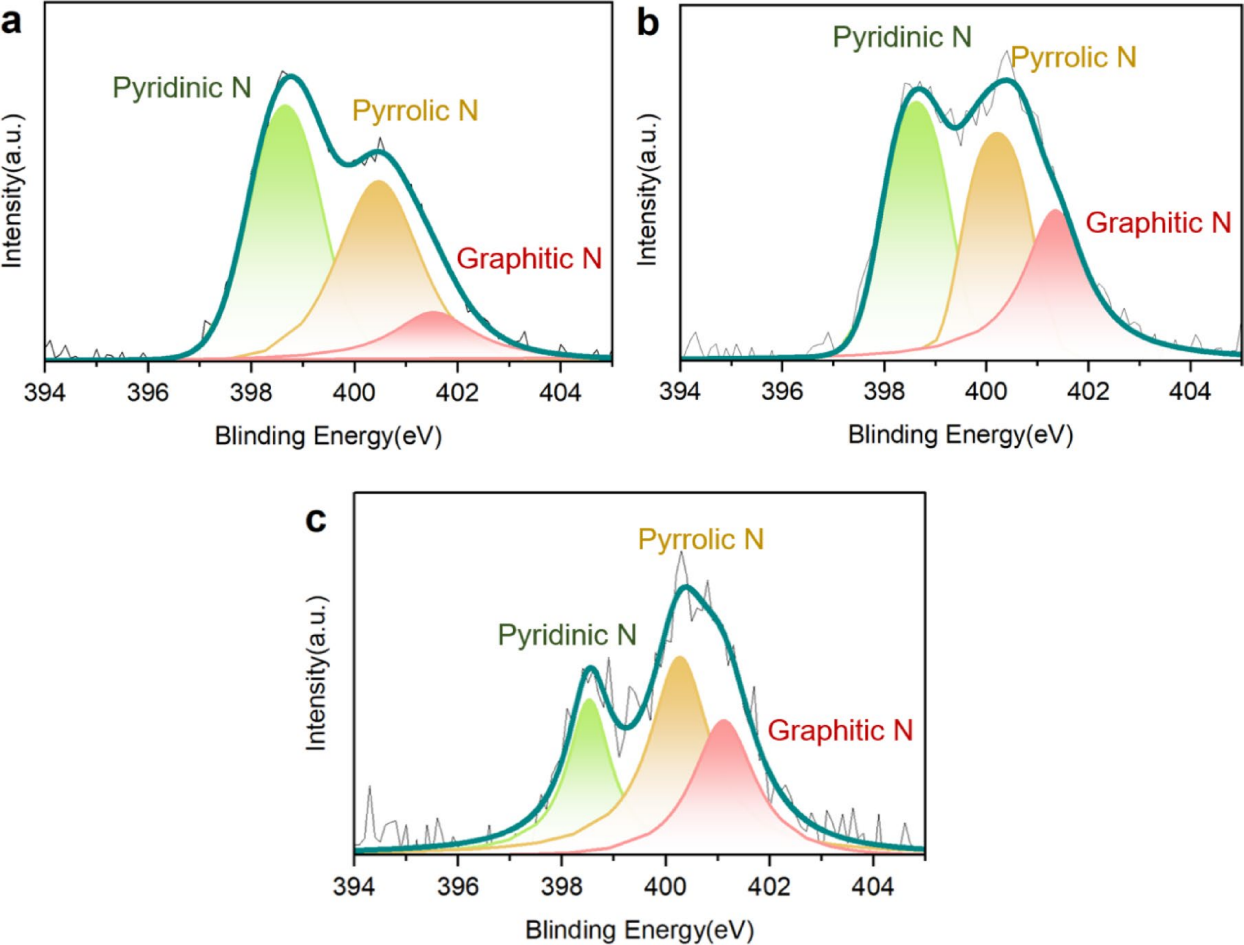
XPS N1s spectra of (**a**) NAC-600, (**b**) NAC-750 and (**c**) NAC-900.

As shown in Fig. 4, the deconvolution of the N 1s spectra further elucidates the evolution of N functional groups with an increase in N-doping temperature. Specifically, the peak at 398.4 eV corresponds to pyridinic N, the peak at 400.0 eV is assigned to pyrrolic N, and the peak at 401.0 eV represents graphitic N^23,24^. The atomic percentages of these N species in the NAC samples are summarized in Table 3. With the increase in temperature, the content of pyridinic N decreases from 3.57 at% in NAC-600 to 2.05 at% in NAC-750, and further to 0.84 at% in NAC-900. A similar trend is observed for pyrrolic N, which decreases from 3.05 at% in NAC-600 to 1.81 at% in NAC-750, and then to 1.44 at% in NAC-900. In contrast, the content of graphitic N increases from 1.05 at% in NAC-600 to 1.68 at% in NAC-750, followed by a decline to 0.85 at% in NAC-900. These variations can be attributed to the different thermal stabilities of nitrogen species in carbonaceous materials. Pyridinic and pyrrolic N generally exhibit lower thermal stability than graphitic N. As the doping temperature rises, the less stable species tend to decompose or undergo structural rearrangement, partially converting into graphitic N. This transformation explains the elevated graphitic N content in NAC-750 compared to NAC-600. However, further increasing the temperature to 900 °C results in the decomposition and volatilization of nitrogen species, likely released in the form of gaseous products (e.g., NOx or HCN), leading to an overall decline in the total N content, including graphitic N.

**Table 3:** Atomic percentage of N functional groups in the ACs.

Sample	NAC-600 (at%)	NAC-750 (at%)	NAC-900 (at%)
Pyridinic N	3. 57	2. 05	0. 84
Pyrrolic N	3. 05	1. 81	1. 44
Graphitic N	1. 05	1. 68	0. 85

**Fig. 5:**
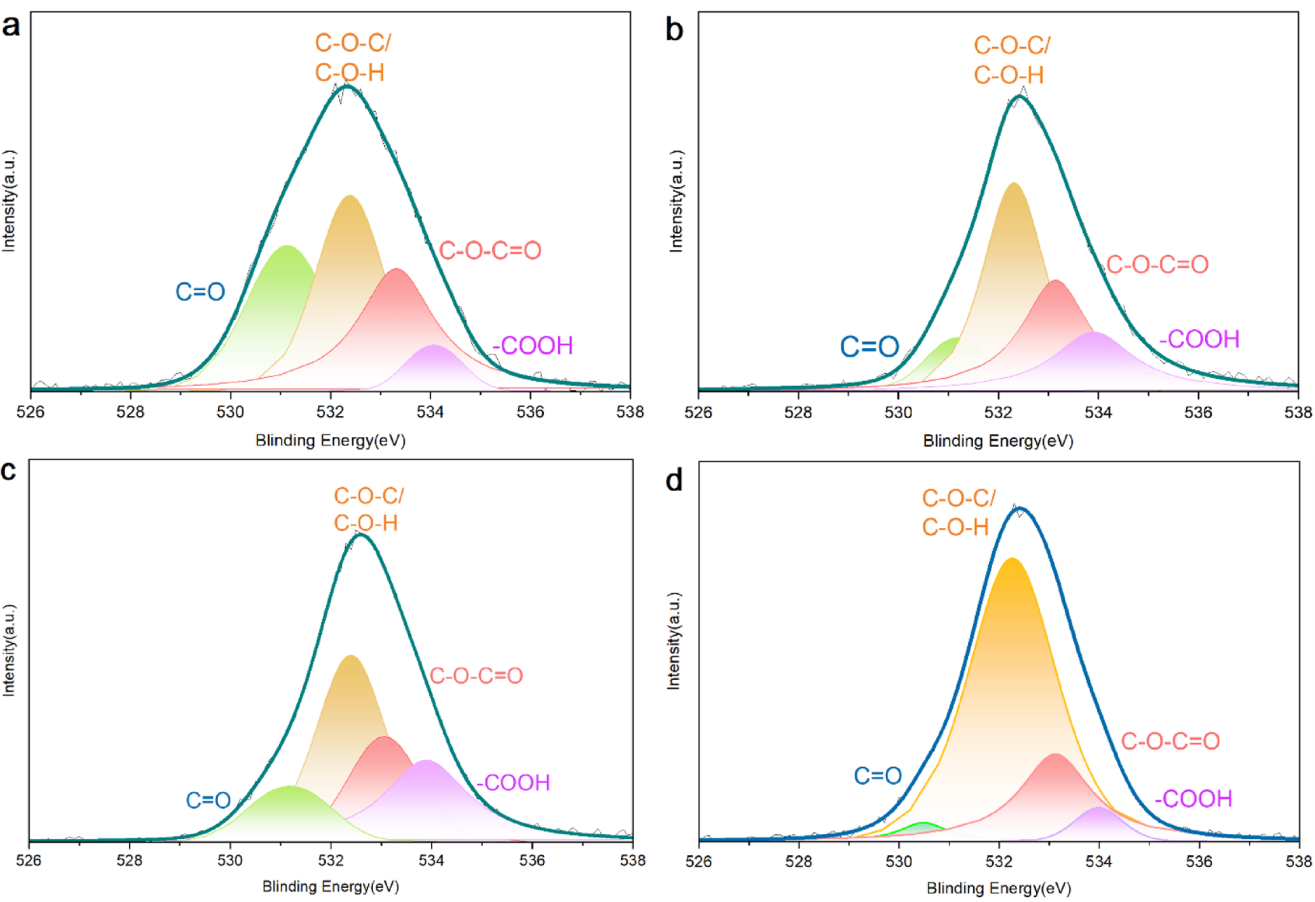
XPS O 1s spectra of (**a**) AC, (**b**) NAC-600, (**c**) NAC-750 and (**d**) NAC-900.

The O 1s peak fitting results for the AC samples are presented in Fig. 5. The peak centred at (531.1 ± 0.2) eV is assigned to oxygen atoms in carbonyl groups (C=O). The peak at (532.3 ± 0.2) eV corresponds to oxygen in ether (C–O–C) or hydroxyl (C–O–H) groups, while the peak at (533.1 ± 0.2) eV is attributed to ester and anhydride groups (C–O). The peak at (533.8 ± 0.2) eV is ascribed to oxygen in carboxyl groups (O=C–OH)^25,26^. As summarized in Table 4, the pristine AC exhibits the highest total content of O functional groups, including carbonyl (3.12 at%), ether/hydroxyl (3.43 at%), and ester/anhydride (3.30 at%), along with a relatively low carboxyl content (0.64 at%). These features are mainly derived from the inherent O-containing species in the carbon precursor (i.e., coal). Upon N-doping at 600 °C, a notable reduction in the content of carbonyl, ether/hydroxyl, and ester/anhydride groups is observed in NAC-600 (e.g., carbonyl decreases from 3.12 at% to 0.69 at%), which can be attributed to partial thermal decomposition or substitution by newly introduced N-containing species. With an increase in temperature to 750 °C, further transformation of O functional groups occurs. Although the content of ether/hydroxyl and ester/anhydride groups in NAC-750 remains lower than that in pristine AC (1.95 at% vs. 3.43 at% and 2.44 at% vs. 3.30 at%, respectively), a significant increase in carbonyl content is observed, reaching 3.39 at%, which exceeds that of NAC-600 (0.69 at%). This trend can be explained by the difference in thermal stabilities of O functional groups, following the order: of ether > carbonyl > hydroxyl > carboxyl. As the temperature increases, the less thermally stable groups (e.g., hydroxyl and carboxyl) tend to decompose or convert into more stable forms such as carbonyl groups. At a further elevated temperature of 900 °C, the NAC-900 sample shows a distinctive ether content (6.93 at%), significantly higher than that in NAC-750 (1.95 at%). Simultaneously, the atomic percentage of other O functional groups decreases, indicating their transformation into ether groups, which are the most thermally stable among them. These results suggest that the N-doping temperature not only governs the incorporation of N functional groups but also influences the composition of O-containing species on ACs.

**Table 4:** Atomic percentage of O functional groups in the AC samples.

Sample	AC (at%)	NAC-600 (at%)	NAC-750 (at%)	NAC-900 (at%)
C=O	3. 12	0. 69	3. 39	0. 35
C–O–C/C–O–H	3. 43	2. 72	1. 95	6. 93
C–O–C=O	3. 30	2. 14	2. 44	2. 36
–COOH	0. 64	1. 34	1. 15	0. 44

**Fig. 6:**
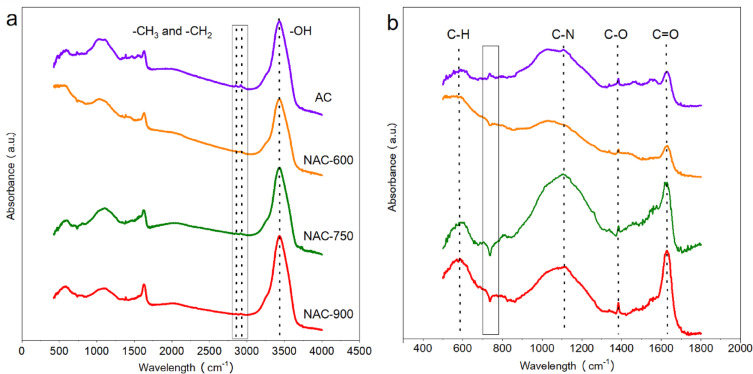
FT-IR spectra of the ACs.

The FT-IR spectra of ACs are presented in Fig. 6. A broad and intense absorption band centred around ~ 3440 cm^−1^ is observed, corresponding to the stretching vibrations of hydroxyl (–OH) groups from alcohols and phenols, as well as adsorbed water^27^. The area of this peak decreases with increasing N-doping temperature (i.e., AC > NAC-600 > NAC-750 > NAC-900), indicating a reduction in surface hydroxyl content due to thermal degradation and dehydroxylation at elevated temperatures. The features at ~ 2920 cm^−1^ and ~ 2850 cm^−1^ are assigned to the asymmetric and symmetric stretching vibrations of aliphatic C–H bonds (–CH3, –CH_2_–)^18^. The decreasing intensity of these peaks with higher temperatures indicates partial decomposition or structural rearrangement of aliphatic groups. The absorption band at ca. 1110 cm^−1^, attributed to C–O stretching vibrations from ether (C–O–C), alcohol, or phenolic groups^28^, shows a slight reduction in intensity for NAC-600 compared to AC. However, in samples NAC-750 and NAC-900, this peak intensity significantly increases, exceeding that of AC. The weak adsorption peak at 1400 cm^−1^ represents the C–O and/or C=O stretching vibration. A similar trend is observed for the C=O stretching vibration at ~ 1630 cm^−1^, which shows a marked increase in NAC-750 and NAC-900 relative to AC^29,30^. These changes in the functional group acquired by FTIR are consistent with the XPS results.

**Fig. 7:**
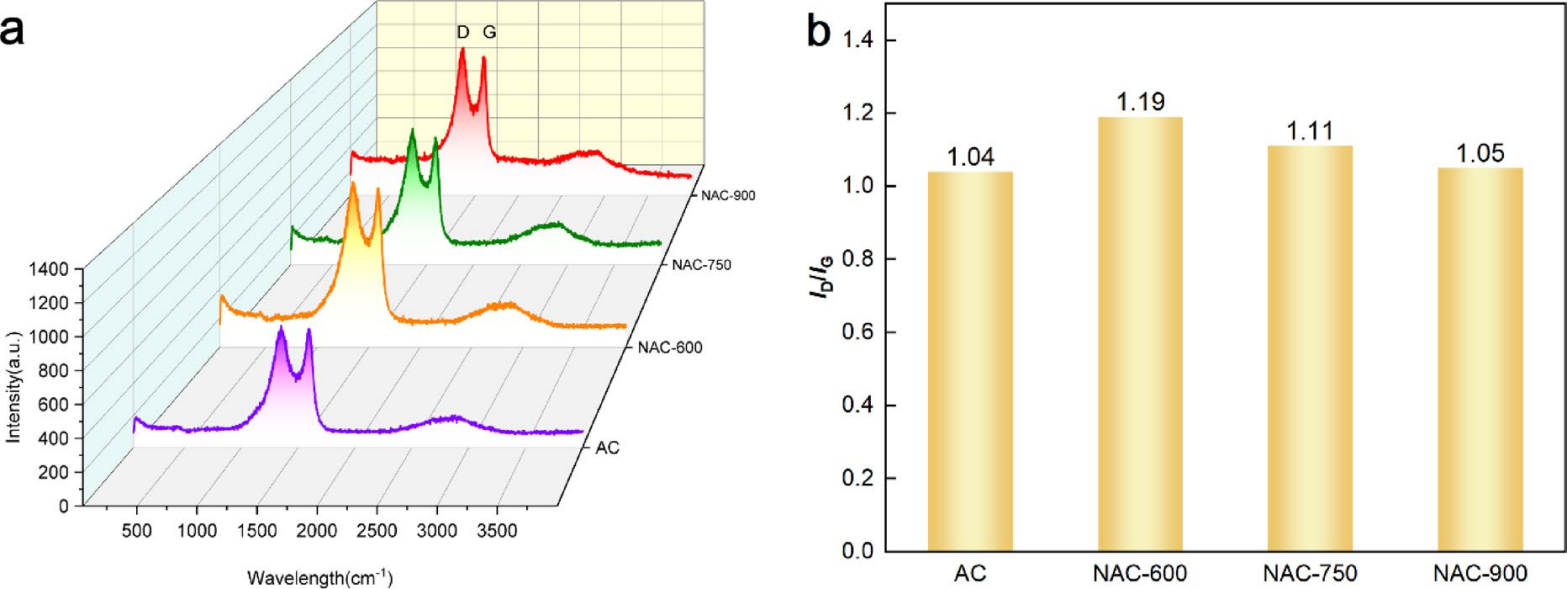
(**a**) Raman spectra (532 nm) of the ACs and (**b**) *I*_D_/*I*_G_ calculated from the ratio of D and G peak intensity.

The Raman spectra of the ACs, as shown in Fig. 7, exhibit distinct D and G bands centred at approximately 1350 cm^−1^ and 1575 cm^−1^, respectively. The degree of graphitization in carbon materials is commonly evaluated using the* I*_D_*/I*_G_ ratio acquired from Raman spectra, where the G band (ca. 1575 cm^−1^) corresponds to *sp*^2^-bonded carbon atoms in hexagonal graphitic rings, while the D band (ca. 1350 cm^−1^) arises from *sp*^3^-bonded carbon atoms associated with structural defects, edge imperfections, and lattice distortions. After N-doping treatment, the NAC samples exhibit a higher* I*_D_*/I*_G_ ratio compared to the pristine AC, indicating the formation of more disordered carbon structures due to the introduction of non-*sp*^2^ structures via co-pyrolysis with melamine. As the N-doping temperature increases, the *I*_D_*/I*_G_ ratio decreases from 1.19 for NAC-600 to 1.05 for NAC-900, suggesting a progressive reduction in structural defects and an enhancement in graphitic degree. This trend implies that elevated treatment temperatures promote defect healing and facilitate graphitization, thereby improving the crystalline order within the carbon microstructure.

### CO_2_ adsorption performances of the ACs

Adsorption performances of CO_2_ at concentrations of 10, 15, 20, and 30 vol% were evaluated using a dynamic vapour sorption (DVS) instrument under ambient conditions (25 °C, 1 bar). The CO_2_ curves of adsorption capacity versus time are presented in Fig. S2 (i.e., the *q*-t curves). The value $q_{{{\text{m}},{\text{CO}}_{{2}} }}$ was measured from the CO_2_ adsorption capacity (i.e., mg/g) at the time when the adsorption was nearly saturated. To further understand the CO_2_ adsorption behavior of activated carbons (ACs), the experimental data were fitted using both Langmuir and Freundlich isotherm models. Nonlinear regression was performed to obtain the corresponding model parameters and correlation coefficients (*R*^2^), as shown in Fig. S3 and Table S1. All samples exhibited *R*^2^ values above 0.99, indicating that both models provide an excellent fit to the experimental data, with the Freundlich model showing slightly better agreement overall. This suggests that CO_2_ adsorption on these ACs involves monolayer adsorption on heterogeneous surfaces, rather than perfectly uniform adsorption sites. Among the four samples, AC, NAC-600, and NAC-750 showed comparable adsorption capacities, whereas NAC-900 exhibited a lower Freundlich constant (*K*_F_), consistent with its reduced CO_2_ uptake. Notably, the Freundlich exponent (n) of NAC-900 decreased significantly, indicating increased surface heterogeneity. This observation aligns with our earlier characterization results, which showed micropore collapse and partial pore widening due to high-temperature treatment at 900 °C. Overall, the results confirm that moderate nitrogen doping temperatures (600–750 °C) enhance CO_2_ adsorption performance by slightly increasing capacity while maintaining high adsorption affinity.

**Fig. 8:**
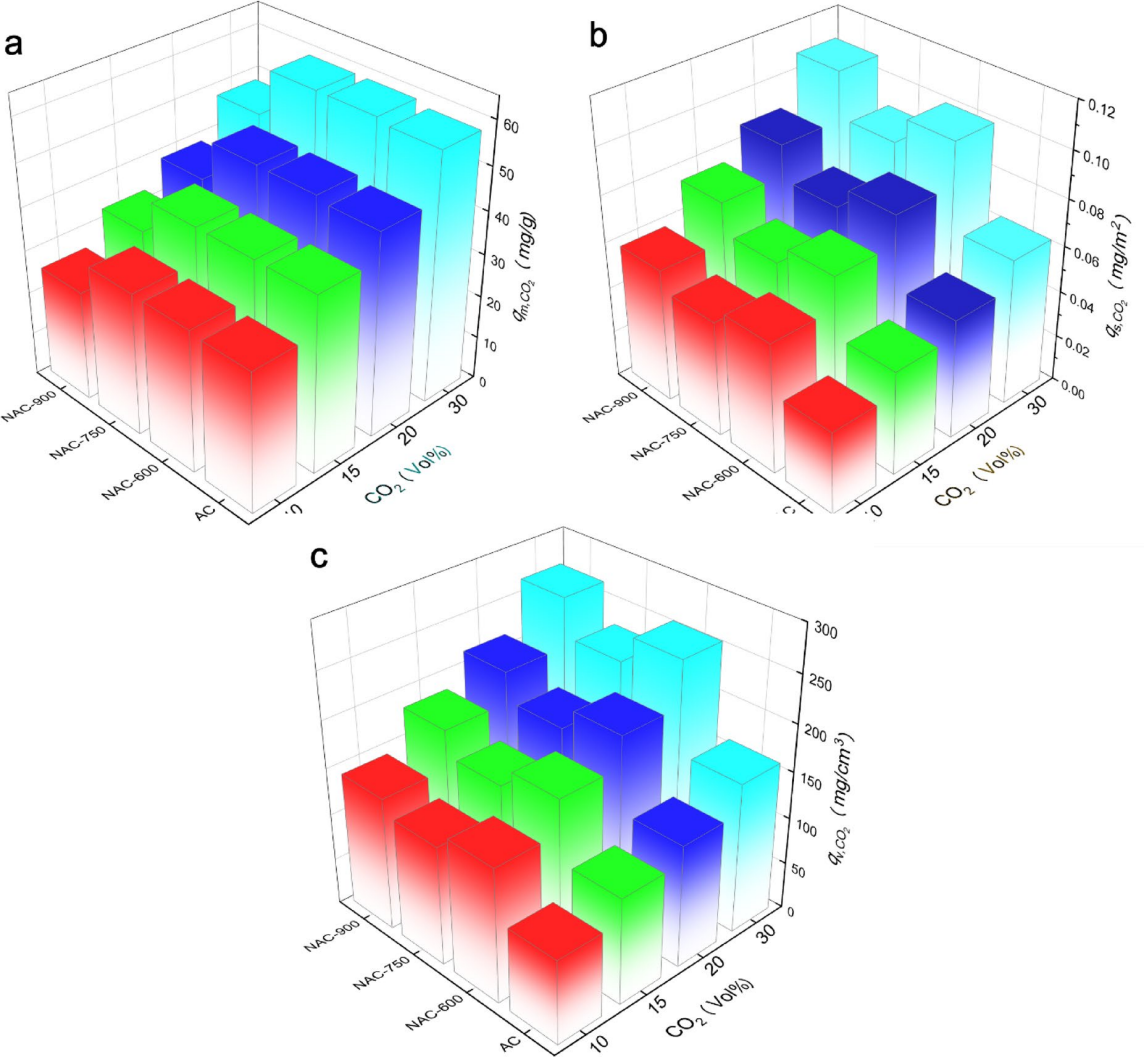
(**a**) CO_2_ adsorption capacity acquired by DVS and (**b**) CO_2_ adsorption capacity per unit microporous surface area and (c) per unit micropore volume of corresponding AC adsorbent.

As shown in Fig. 8a, $q_{{{\text{m}},{\text{CO}}_{{2}} }}$ increases with an increase in the concentration of CO_2_. Notably, the $q_{{{\text{m}},{\text{CO}}_{{2}} }}$ NAC-750 and NAC-600 are highly similar across all tested CO_2_ concentrations selected in this study, reaching up to 58 mg/g at 30 vol% CO_2_, being slightly higher than that of the pristine AC. Although NAC-750 and NAC-600 display lower *S*_BET_ and *V*_mic_ compared to the pristine AC, their CO_2_ adsorption remains comparable, which can be tentatively attributed to the introduction of N functional groups onto the AC surfaces, enhancing the affinity toward CO_2_ molecules. NAC-900 exhibited the lowest $q_{{{\text{m}},{\text{CO}}_{{2}} }}$, mainly due to its significantly lower *S*_BET_ and *V*_mic_ than other ACs. To further analyze the influence of surface chemistry independently from porosity, $q_{{{\text{s}},{\text{CO}}_{{2}} }}$ and $q_{{{\text{v}},{\text{CO}}_{{2}} }}$ were calculated using Eqs. (1) and (2) in this study.

**Fig. 9.**
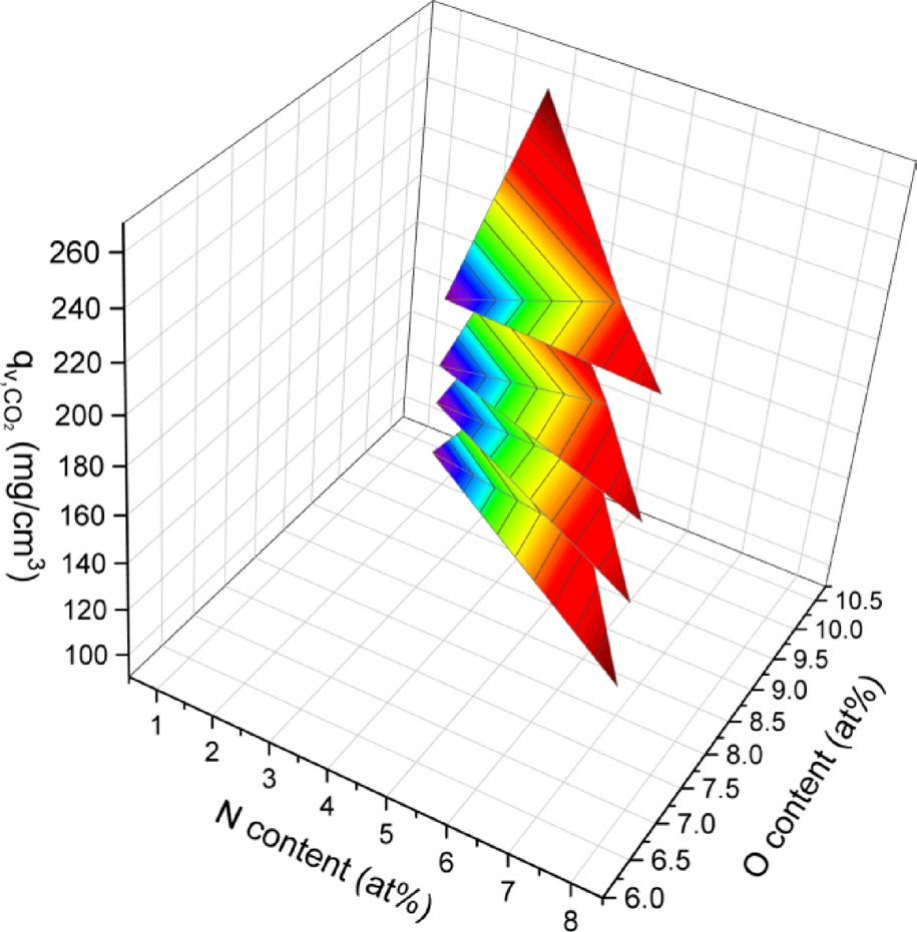
3D correlation mapping between the N and O functional group contents of the ACs and their $q_{{{\text{v}},{\text{CO}}_{2} }}$.

As shown in Fig. 8b and c, both $q_{{{\text{s}},{\text{CO}}_{{2}} }}$ $q_{{{\text{v}},{\text{CO}}_{{2}} }}$ the NAC samples are notably higher than those of the pristine AC, proving that the N functionalization enables to improvement of the affinity between carbon adsorbents and CO_2_. With an increase in N doping temperature from 600 °C to 900 °C, both $q_{{{\text{s}},{\text{CO}}_{{2}} }}$ and $q_{{{\text{v}},{\text{CO}}_{{2}} }}$ initially decreased and then increased. The simultaneous influence of N and O content $q_{{{\text{v}},{\text{CO}}_{{2}} }}$ is illustrated in the three-dimensional plot in Fig. 9. It can be inferred that an N content of 2–8 at% along with a moderate O content (e.g., 8–11 at%) is favourable for the affinity between carbon surface and CO_2_ molecules. Conversely, high O content together with a rare N content exhibit no beneficial influence on $q_{{{\text{v}},{\text{CO}}_{{2}} }}$, suggesting a synergistic promotion effect arising from a balanced composition of N and O functional groups. These results highlight the importance of optimizing heteroatom doping to tailor surface chemistry for improved CO_2_ adsorption performance.

As shown in Fig. 8c, NAC-900 exhibits the highest $q_{{{\text{v}},{\text{CO}}_{{2}} }}$ among the samples. Although its absolute content of pyrrolic-N (1.44 at%) is lower than that in NAC-600, NAC-900 shows a relatively higher proportion of pyrrolic-N among the total nitrogen species, along with the highest content of C–O–C/C–O–H groups (6.93 at%). These oxygen-containing groups can enhance CO_2_ adsorption through dipole–quadrupole interactions and hydrogen bonding. Furthermore, pyrrolic-N is known to provide electron-rich sites that favor Lewis acid–base interactions with CO_2_ molecules, thereby contributing to chemisorption^31^. The synergistic effect of surface functionalities and improved structural order thus accounts for the superior adsorption performance of NAC-900. In comparison, although NAC-750 contains a moderate level of pyrrolic N (1.81 at%), it $q_{{{\text{v}},{\text{CO}}_{{2}} }}$ remains lower than that of NAC-600 and NAC-900, likely due to the relatively weak affinity of carbonyl groups toward CO_2_ molecules compared to other O functional groups such as hydroxyl and carboxyl groups^32^. Notably, NAC-600, which possesses the highest pyrrolic-N content (3.05 at%), achieves a $q_{{{\text{v}},{\text{CO}}_{{2}} }}$ value comparable to that of NAC-900. The slightly higher $q_{{{\text{v}},{\text{CO}}_{{2}} }}$ for NAC-900 than NAC-600 can be attributed to the higher ether/hydroxyl groups of the former, introducing the extra dipole–quadrupole interactions with CO_2_ molecules. Above all, NAC-600 and NAC-900, both nitrogen-doped samples, exhibit comparably high $q_{{{\text{v}},{\text{CO}}_{{2}} }}$, surpassing that of the pristine AC. This enhanced performance can be attributed to the balanced distribution of nitrogen and oxygen functional groups on natural-sourced ACs, especially the co-existence of pyrrolic nitrogen, ether (C–O–C), and hydroxyl (C–O–H) groups.

### H_2_O adsorption performances of the ACs

**Fig. 10:**
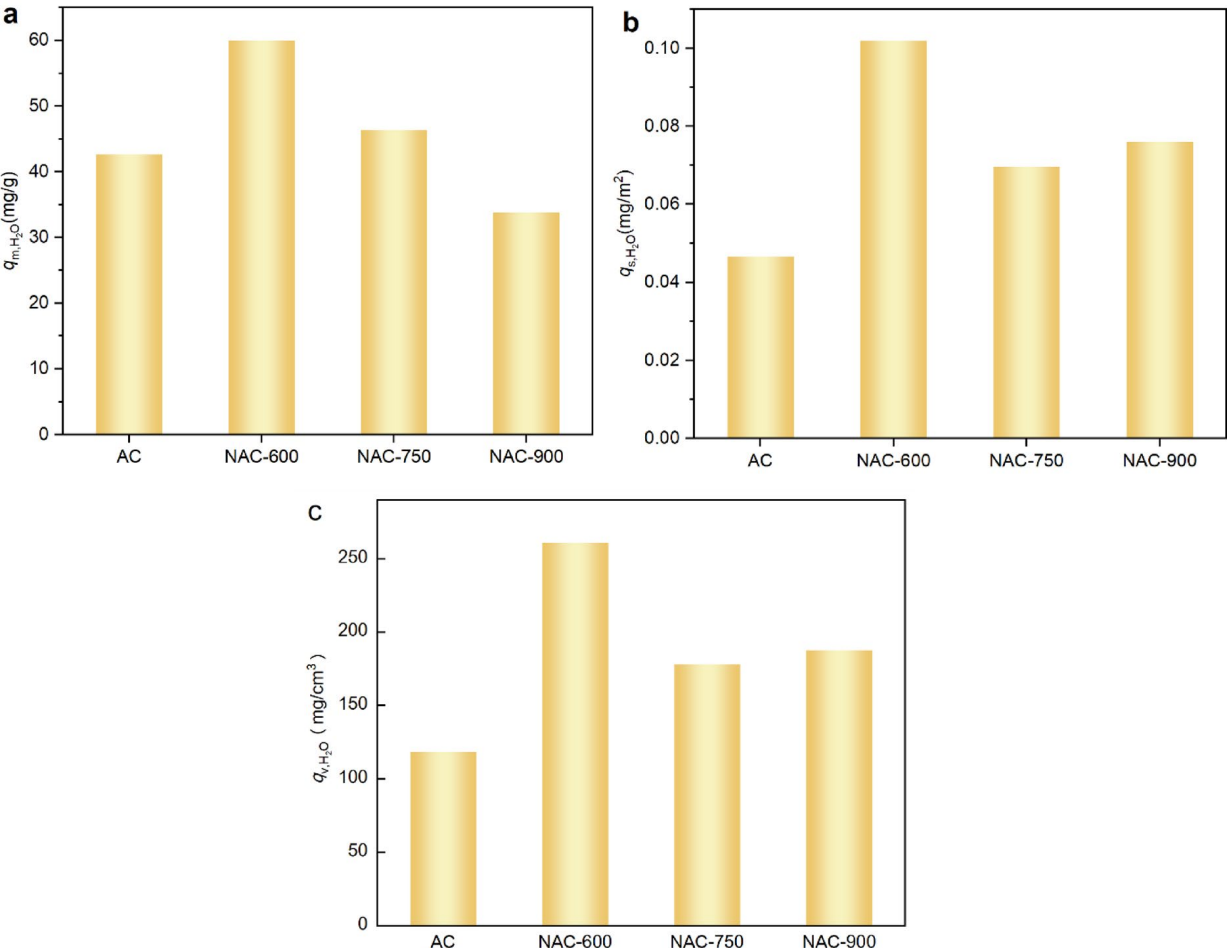
(**a**) Adsorption capacity of H_2_O per unit mass ($q_{{{\text{m}},{\text{H}}_{{\text{2}}} {\text{O}}}}$); (**b**) H_2_O adsorption capacity per unit microporous surface area ($q_{{{\text{s}},{\text{H}}_{{\text{2}}} {\text{O}}}}$); (**c**) H_2_O adsorption capacity per unit micropore volume ($q_{{{\text{v}},{\text{H}}_{{\text{2}}} {\text{O}}}}$).

In practical carbon capture applications, CO_2_ in flue gas typically coexists with moisture. Therefore, the moisture adsorption performances (at 20 RH%) of both pristine and N-doped ACs were evaluated, with the corresponding *q*-*t* profiles presented in Fig. S4. As shown in Fig. 10a, despite the N-doped samples exhibiting relatively lower *S*_BET_ and *V*_mic_ compared to pristine AC, NAC-600 and NAC-750 still demonstrate significantly higher adsorption capacity of moisture over 40 min (i.e., $q_{{{\text{m}},{\text{H}}_{{2}} {\text{O}}}}$ 59.9 mg/g and 46.3 mg/g, respectively, *vs.* 42.5 mg/g for pristine AC). In contrast, NAC-900, which possesses a 44.3% lower *S*_BET_ area than the pristine AC, shows a notably reduced $q_{{{\text{m}},{\text{H}}_{{2}} {\text{O}}}}$ of 33.8 mg/g. To decouple the influence of porosity from surface chemistry, and $q_{{{\text{v}},{\text{H}}_{{2}} {\text{O}}}}$ were calculated and shown in Fig. 10b and c, respectively. The trends in $q_{{{\text{s}},{\text{H}}_{{2}} {\text{O}}}}$ and $q_{{{\text{v}},{\text{H}}_{{2}} {\text{O}}}}$ are consistent across all samples, i.e., NAC-600 exhibits the highest values, followed by NAC-900 and NAC-750. In general, both $q_{{{\text{s}},{\text{H}}_{{2}} {\text{O}}}}$ and $q_{{{\text{v}},{\text{H}}_{{2}} {\text{O}}}}$ of the NAC samples are higher than that of the pristine AC, illustrating the positive effect of N doping on H_2_O adsorption.

Among oxygen-containing surface functionalities, hydroxyl (–OH), carboxyl (–COOH), and ether (C–O–C) groups are widely recognized for their strong affinity toward water molecules due to their capacity to form hydrogen bonds. Notably, NAC-600 exhibits the highest carboxyl group content (1.34 at%), a functional group known for its strong hydrophilicity and effectiveness in enhancing water uptake. In contrast, NAC-900 features a significantly high content of ether and hydroxyl groups (C–O–C/C–O–H: 6.93 at%), also being beneficial for its $q_{{{\text{s}},{\text{H}}_{{2}} {\text{O}}}}$ and $q_{{{\text{v}},{\text{H}}_{{2}} {\text{O}}}}$. These findings are in agreement with previous studies regarding the enhancement of O functional groups on moisture adsorption capacity by ACs^33^. Previous reports have also confirmed the enhancing effect of N functional groups, primarily by strengthening electrostatic interactions and hydrogen bonding with water molecules^34^. Pyridinic and pyrrolic N species are generally more hydrophilic than graphitic nitrogen due to their electron-rich lone pairs, which facilitate favourable interactions with water molecules. NAC-600 again demonstrates the highest content of both pyridinic nitrogen (3.57 at%) and pyrrolic nitrogen (3.05 at%), which strongly correlates with its highest $q_{{{\text{s}},{\text{H}}_{{2}} {\text{O}}}}$ and $q_{{{\text{v}},{\text{H}}_{{2}} {\text{O}}}}$ among ACs. Thus, the best $q_{{{\text{v}},{\text{H}}_{{2}} {\text{O}}}}$ of NAC-600 can be attributed to the synergistic presence of hydrophilic O functional groups (such as –COOH and C–O–H) and active N sites (pyridinic and pyrrolic nitrogen). In summary, NAC-600 exhibits nearly the highest $q_{{{\text{v}},{\text{H}}_{{2}} {\text{O}}}}$ and $q_{{{\text{v}},{\text{H}}_{{2}} {\text{O}}}}$ among all AC samples, indicating its optimal surface chemistry for CO_2_ and H_2_O adsorption.

### Toluene adsorption performances of the ACs

**Fig. 11:**
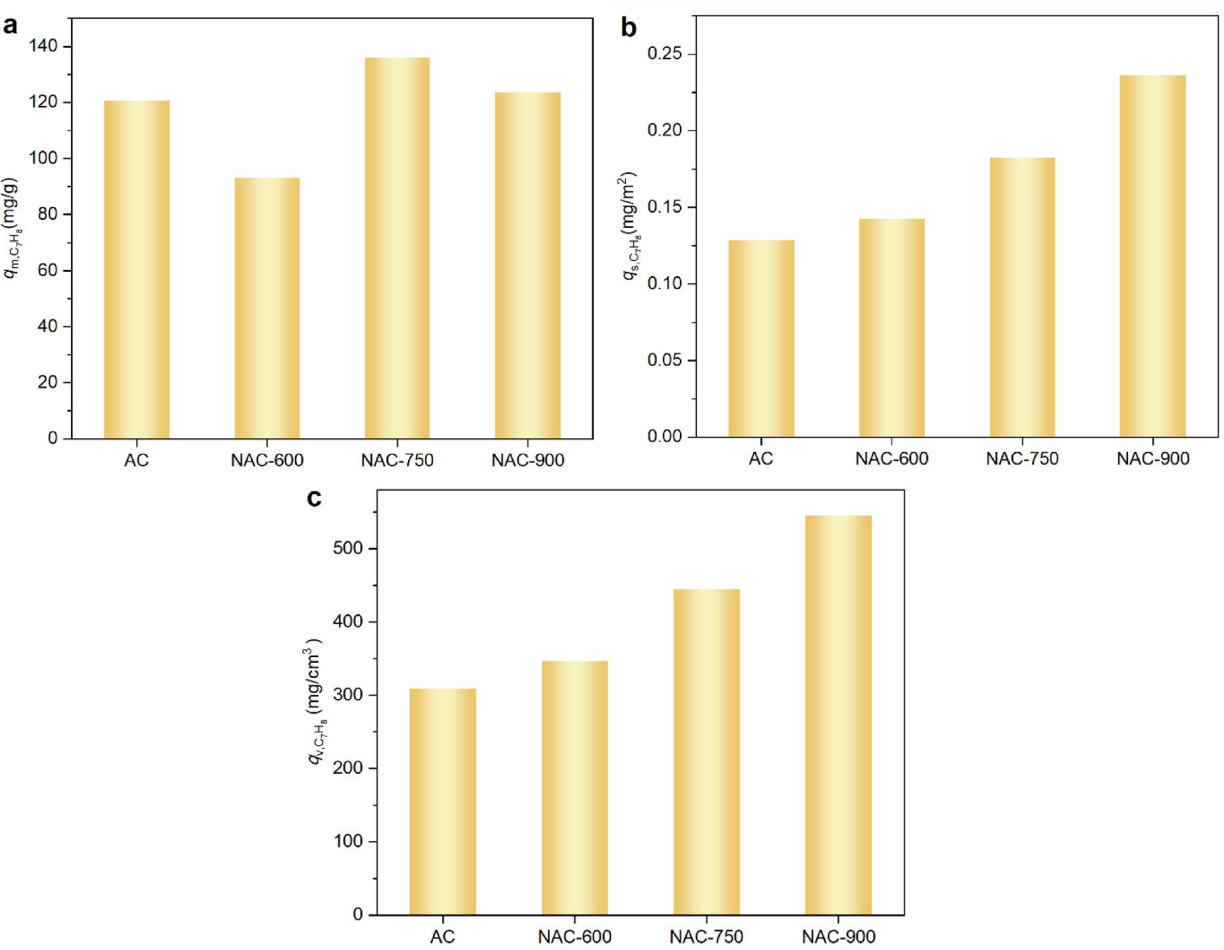
(**a**) Adsorption capacity of toluene per unit mass ($q_{{{\text{m}},{\text{C}}_{{\text{7}}} {\text{H}}_{{\text{8}}} }}$); (**b**) toluene adsorption capacity per unit microporous surface area ($q_{{{\text{s}},{\text{C}}_{{\text{7}}} {\text{H}}_{{\text{8}}} }}$); (**c**) H_2_O adsorption capacity per unit micropore volume ($q_{{{\text{v}},{\text{C}}_{{\text{7}}} {\text{H}}_{{\text{8}}} }}$).

As one of the most representative coal-fired VOCs in flue gas, toluene exhibits a relatively larger kinetic size than CO_2_ and H_2_O, as well as a much lower molecular polarity. The adsorption *q*-*t* profiles of toluene on the AC samples are presented in Fig. S5. As shown in Fig. 11a, compared to the pristine AC, NAC-600 displays a slightly lower toluene adsorption capacity over 250 min ($q_{{{\text{m}},{\text{C}}_{{7}} {\text{H}}_{{8}} }}$ = 93 mg/g *vs.* 120.7 mg/g). In contrast, both NAC-750 and NAC-900 show significantly higher $q_{{{\text{m}},{\text{C}}_{{7}} {\text{H}}_{{8}} }}$ at 139 mg/g and 125 mg/g, respectively. Therefore, no straightforward correlation can be established between the N or O content in NACs and the $q_{{{\text{m}},{\text{C}}_{{7}} {\text{H}}_{{8}} }}$, as the differences in $q_{{{\text{m}},{\text{C}}_{{7}} {\text{H}}_{{8}} }}$ result from the simultaneous influence of both porosity variation and surface chemistry changes.

After decoupling the effect of porosity, both $q_{{{\text{s}},{\text{C}}_{{7}} {\text{H}}_{{8}} }}$ and $q_{{{\text{v}},{\text{C}}_{{7}} {\text{H}}_{{8}} }}$ were found to increase with an increase in N-doping temperature, as shown in Fig. 11b and c. Specifically, NAC-900 exhibited the highest $q_{{{\text{v}},{\text{C}}_{{7}} {\text{H}}_{{8}} }}$ followed by NAC-750 and NAC-600. This trend correlates with the decreasing *I*_D_/*I*_G_ ratio, from 1.19 (NAC-600) to 1.05 (NAC-900), as illustrated by the Raman data. Higher graphitization degrees are known to facilitate π–π interactions between the delocalized electrons of the graphitic carbon layers and the aromatic rings of toluene molecules, thus enhancing adsorption affinity. In addition, surface chemistry also affects the $q_{{{\text{v}},{\text{C}}_{{7}} {\text{H}}_{{8}} }}$ and $q_{{{\text{ms}},{\text{C}}_{{7}} {\text{H}}_{{8}} }}$. XPS revealed that NAC-900 possesses a higher content of ether or hydroxyl groups and the lowest carboxyl group content (–COOH: 0.44 at%) among the tested samples. The ether groups enriched in NAC-900 are more hydrophobic than carboxyl groups and do not hinder the adsorption of toluene, a molecule with low polarity. In contrast, NAC-600 exhibited higher concentrations of pyridinic N (3.57 at%) and carboxyl groups (–COOH: 1.34 at%). However, pyridinic N may disrupt the conjugated π-system of toluene, reducing the π–π interactions, which can result in a decrease in the $q_{{{\text{v}},{\text{C}}_{{7}} {\text{H}}_{{8}} }}$ of AC adsorbent. Hence, unlike CO_2_ and H_2_O adsorption, NAC-900, prepared at the highest N-doping temperature, exhibits the optimal surface chemistry for toluene adsorption, which is attributed not only to an increased degree of graphitization but also to the rational distribution of functional groups such as graphitic N and ether groups on AC. In addition, as shown in Fig. S6, representative sample NAC-600 maintains over 95% of its initial adsorption capacity after five consecutive adsorption (@ 25 °C)-desorption (@ 120 °C) cycles, indicating its promising reusability and stability.

## Conclusions

In this study, coal-based activated carbons (ACs) with high specific surface area (up to 940 m^2^/g) and micropore volume (up to 0.36 cm^3^/g) were prepared using K_2_CO_3_-assisted physical activation. By adjusting the temperature during the sequential N-doping treatment, the N content in the NACs varied from 1.44 at% to 7.68 at%, while the O content ranged from 6.89 at% to 10.39 at%. The co-pyrolysis with melamine for N doping introduced pore blockage at lower temperatures, such as 600 °C, which can be mitigated by increasing the temperature. For CO_2_ adsorption, NAC samples (especially the NAC-600 and NAC-900) exhibited comparably high $q_{{{\text{v}},{\text{CO}}_{{2}} }}$ values, surpassing that of the pristine AC. This highlights the importance of a balanced N and O functional group composition, especially the co-existence of pyrrolic N, ether (C–O–C), and hydroxyl (C–O–H) groups. NAC-600 demonstrated the highest adsorption capacity of moisture (59.9 mg/g), benefiting from hydrophilic O-groups (such as –COOH, C–O–H) and active N sites (pyridinic and pyrrolic N), making it the optimal surface chemistry for both CO_2_ and H_2_O adsorption. In contrast, NAC-900, which was prepared at the highest N-doping temperature, exhibited the best surface chemistry for toluene adsorption. This enhanced performance is attributed to increased graphitization and rational distribution of graphitic N and ether groups, which promote π–π interactions, favoring toluene adsorption. This study provides valuable insights into enhancing the adsorption performance of multi gas components by simultaneously regulating the N and O functional groups in natural-sourced AC adsorbents.

## Supplementary Information

Below is the link to the electronic supplementary material.

Supplementary Material 1

## Data Availability

The datasets used and/or analysed during the current study available from the corresponding author Zhipeng Qie on reasonable request via qiezhipeng@bjut.edu.cn.
