# Supplementary material for: Engineering nitrogen and oxygen functionalities in naturally sourced activated carbon for multicomponent gas adsorption
Source: Sci Rep. 2025 Aug 1;15:28102. doi: 10.1038/s41598-025-13430-4 (PMC12317000; doi:10.1038/s41598-025-13430-4)
Supplement: Supplementary file 1 — Supplementary Material 1 [file 41598_2025_13430_MOESM1_ESM.docx]

***Supplementary data for***

**Engineering Nitrogen and Oxygen Functionalities in Naturally Sourced Activated Carbon for Multicomponent Gas Adsorption**

Xiupeng Cheng^a^, ^†^, Zhipeng Qie^a, b, †, *^, Huaizhong Xiang^c,d^, Zhongbao Liu^a^, Limingxin Zong^a^, Wenqi He^a^, Xinxin Pi^e^, Hassan Alhassawi^c^, Peiyao Cao^a^, Guang Yang^a^, Shuangshuang Gao^a^

*^a^ College of Mechanical and Energy Engineering, Beijing University of Technology, Beijing 100124, China*

*^b^ Chongqing Research Institute of Beijing University of Technology, Chongqing 401121, China*

*^c^ Department of Chemical Engineering, The University of Manchester, Manchester M13 9PL, UK*

*^d^ Department of Chemistry, Queen Mary University of London, London E1 4NS, UK*

*^e^ College of Mechanical and Electrical Engineering, Qingdao University, Qingdao 266071, China*

Corresponding Author’s email: [qiezhipeng@bjut.edu.cn](mailto:qiezhipeng@bjut.edu.cn) (Z.Q.)

^†^ *These authors contributed equally to this work.*


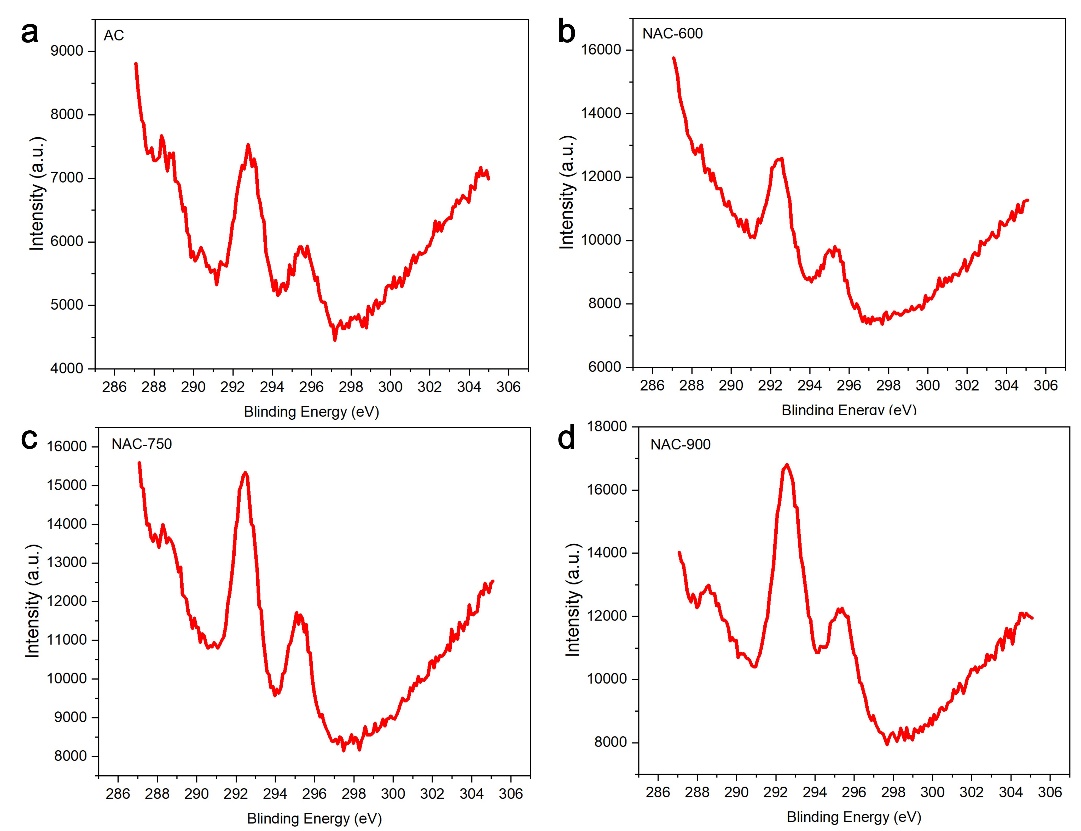


**Fig. S1.** XPS K2p spectra of (a) AC, ( (b) NAC-600, (c) NAC-750 and (d) NAC-900.


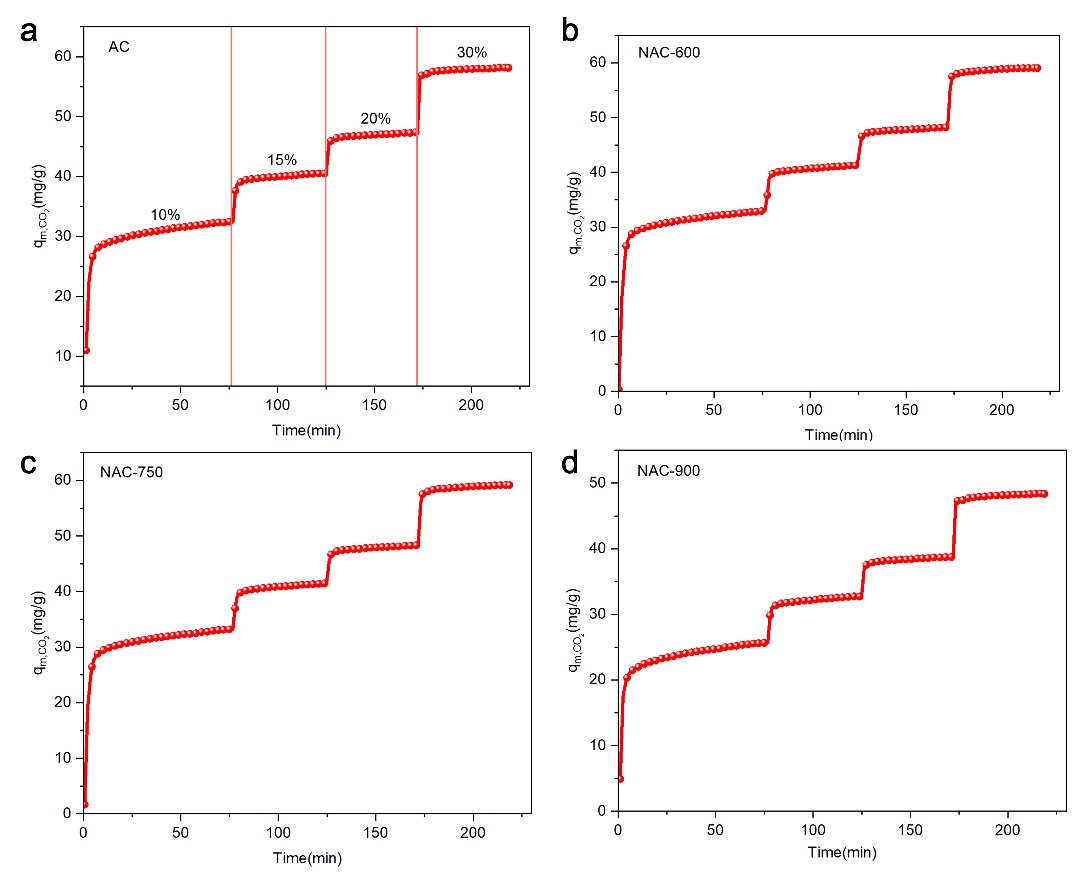


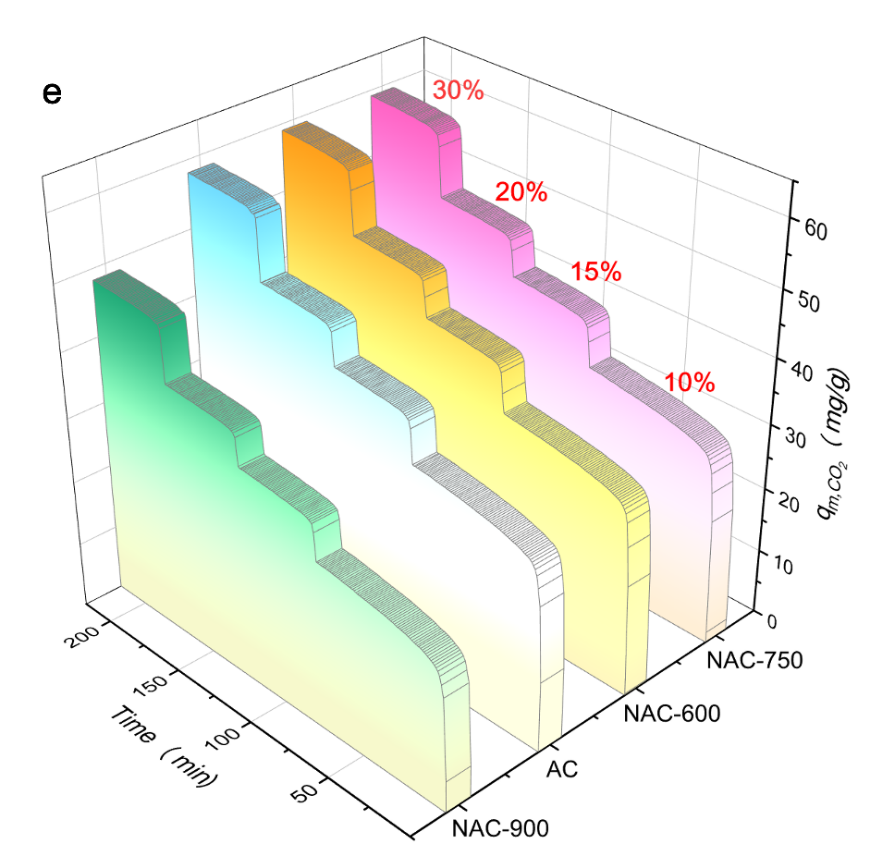


**Fig. S2.** CO_2_ curves of adsorption capacity versus time (a) AC, (b) NAC-600, (c) NAC-750, (d) NAC-900 and (e) 3D comparison chart of .


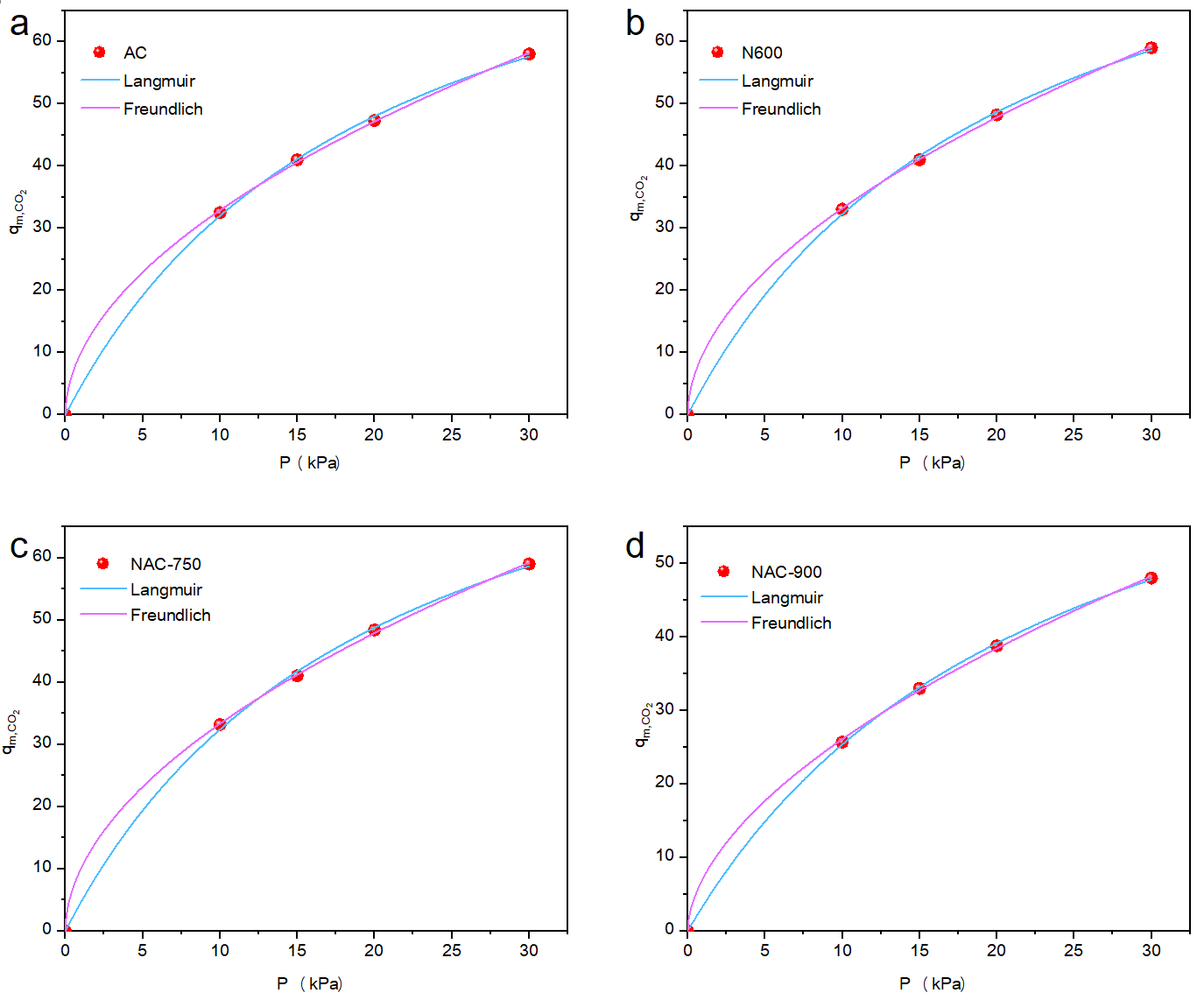


**Fig. S3.** CO_2_ adsorption isotherms of (a) AC, ( (b) NAC-600, (c) NAC-750 and (d) NAC-900.

**Table.S1.** Fitting parameters of CO_2_ adsorption isotherms

|  | Langmuir | | | Freundlich | | |
| --- | --- | --- | --- | --- | --- | --- |
|  | *q*_m_ | *K*_L_ | R^2^ | *K*_F_ | *n* | R^2^ |
| AC | 96 | 0.05 | 0.996 | 9.95 | 1.93 | 0.999 |
| NAC-600 | 98.97 | 0.048 | 0.999 | 9.89 | 1.9 | 0.994 |
| NAC-750 | 98.5 | 0.049 | 0.994 | 9.98 | 1.91 | 0.999 |
| NAC-900 | 85.6 | 0.042 | 0.998 | 7.2 | 1.79 | 0.999 |


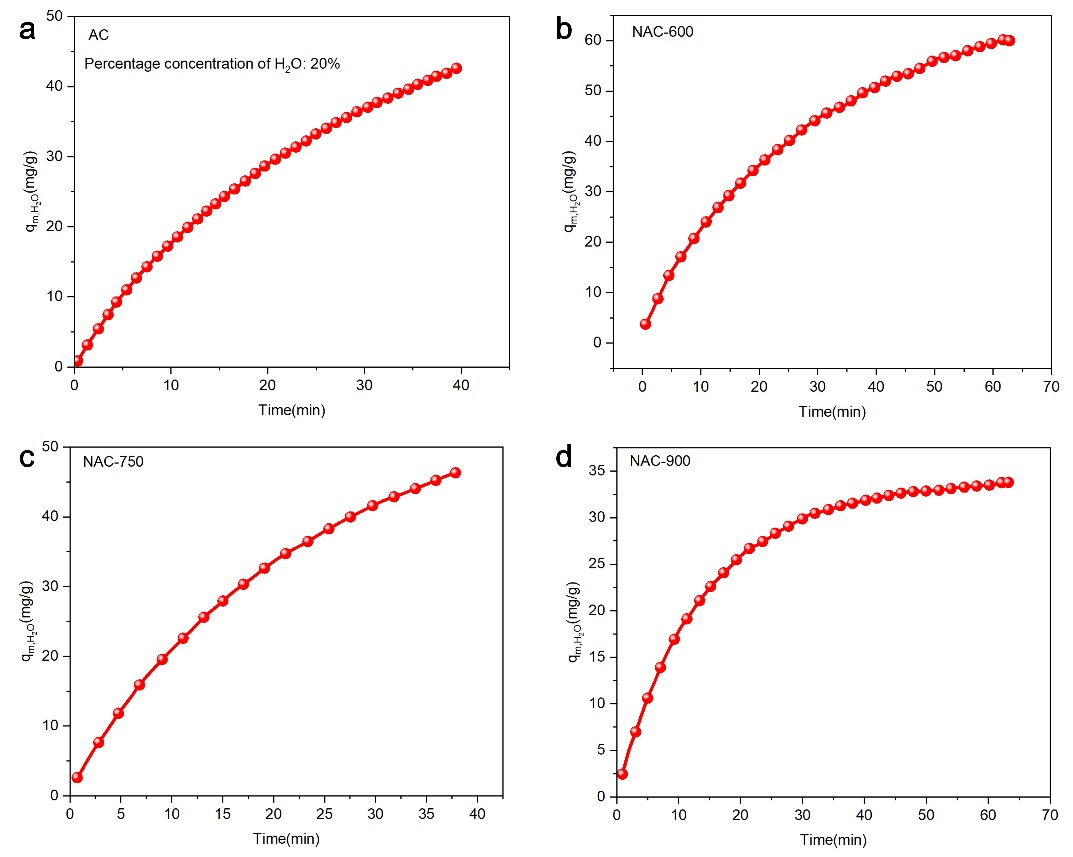


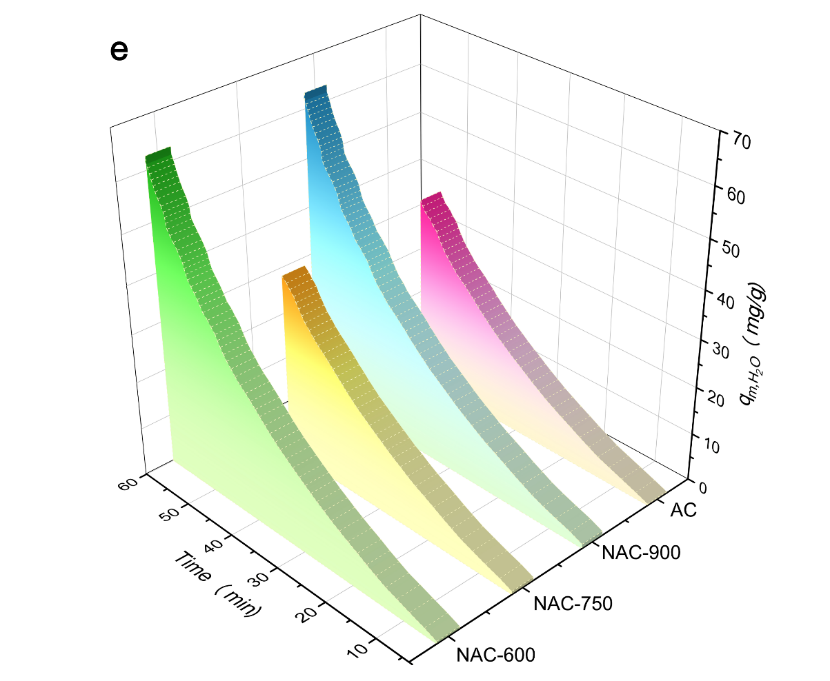


**Fig. S4.** H_2_O curves of adsorption capacity versus time (a) AC, ( (b) NAC-600, (c) NAC-750 ,(d) NAC-900 and(e) 3D comparison chart of .


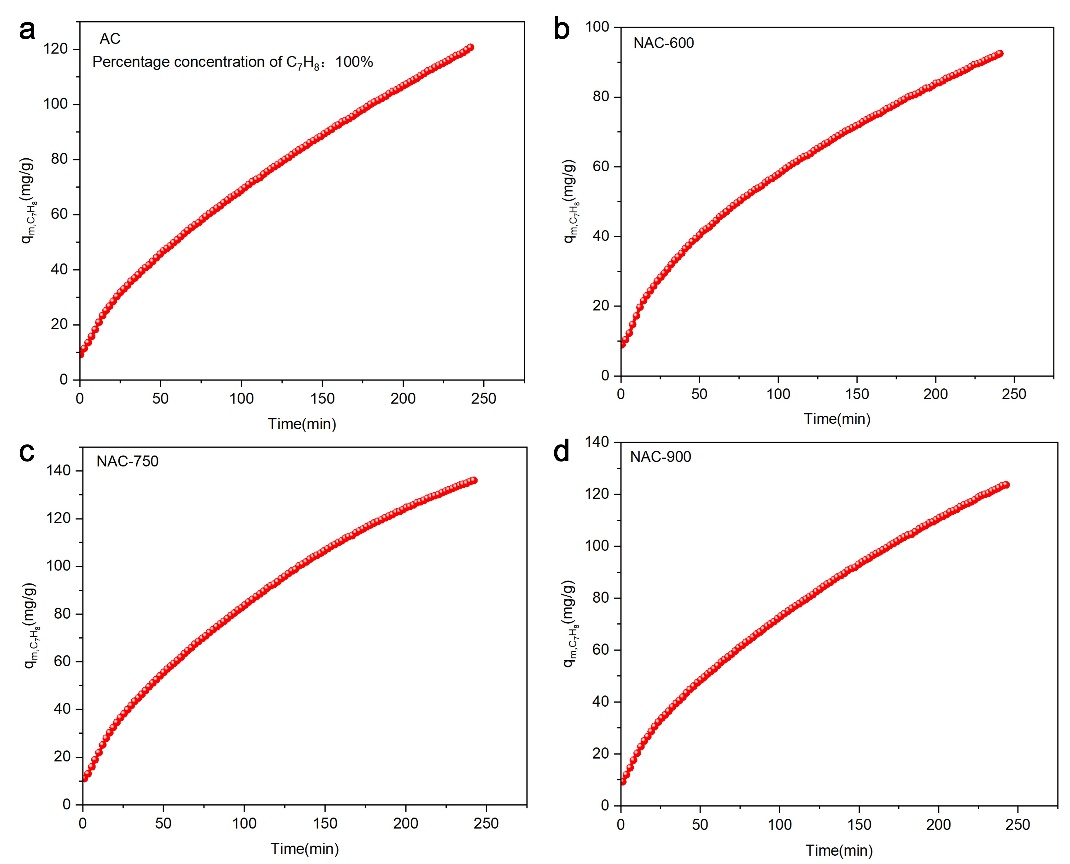


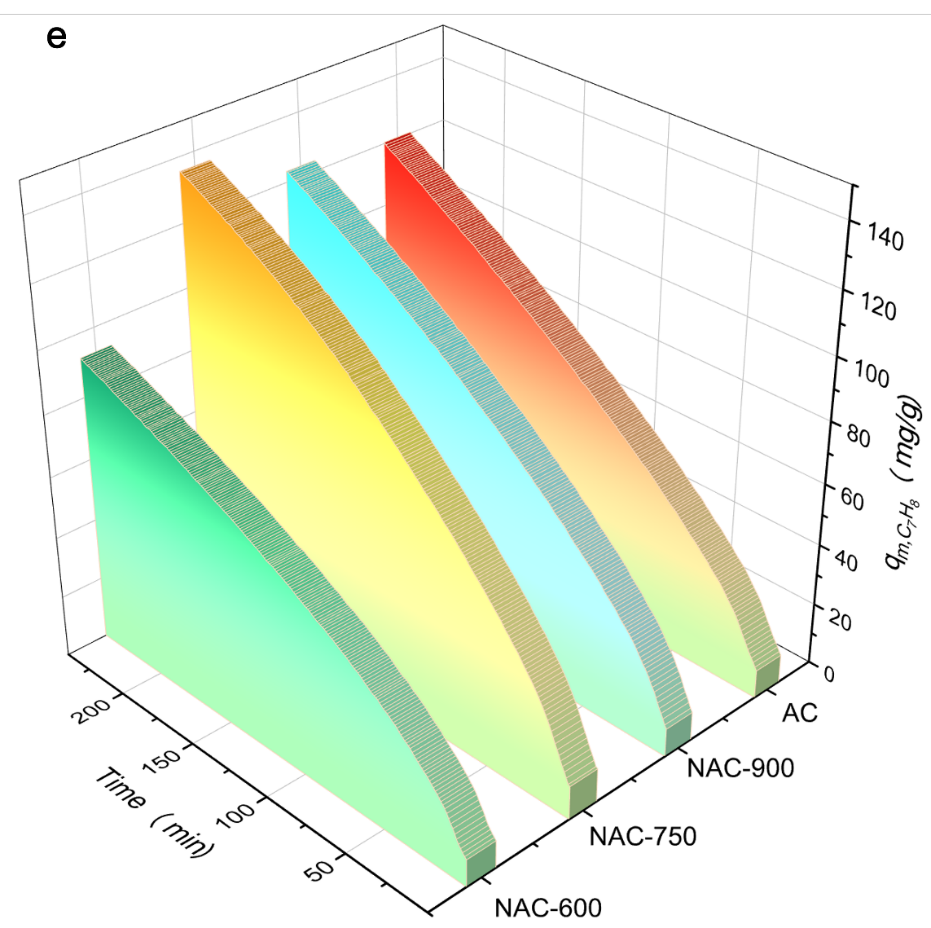


**Fig. S5.** C_7_H_8_ curves of adsorption capacity versus time (a) AC, ( (b) NAC-600, (c) NAC-750 ,(d) NAC-900 and(e) 3D comparison chart of .


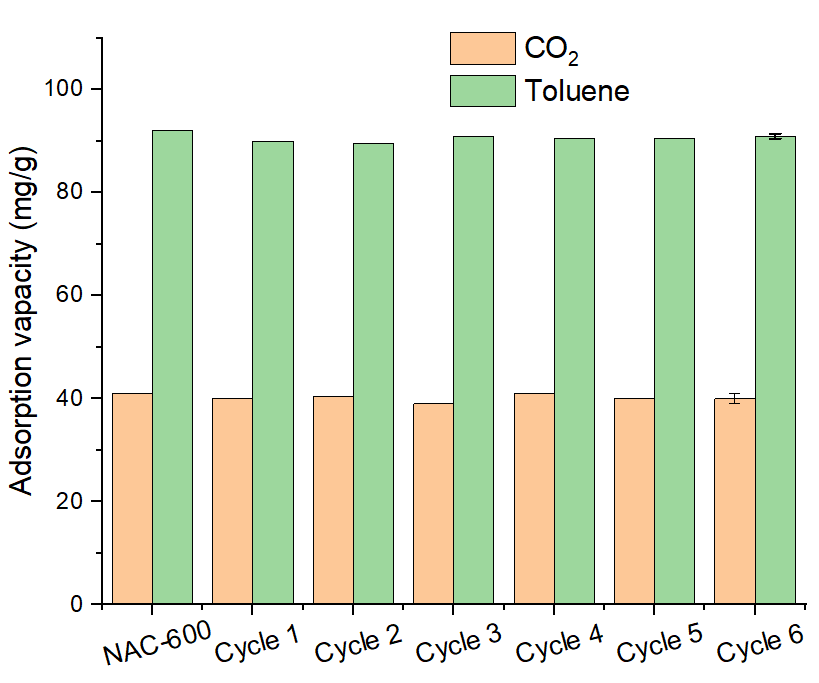


**Fig. S6**. Recyclability of NAC-600 during CO_2_ and toluene adsorption/desorption test
